# Non-syndromic Cleft Lip and Palate Patients of the North Indian Population and the Association of Methylenetetrahydrofolate Reductase Gene

**DOI:** 10.7759/cureus.64812

**Published:** 2024-07-18

**Authors:** Juhi Yadav, Pradeep Raghav, Raj Kumar Mishra, Divya Narain Upadhyaya, Veerendra Prasad, Chandra P Chaturvedi, Kritanjali Singh

**Affiliations:** 1 Department of Orthodontics and Dentofacial Orthopedics, Autonomous State Medical College, Auraiya, Auraiya, IND; 2 Department of Orthodontics, Swami Vivekanand Subharti University, Meerut, IND; 3 Department of Plastic Surgery and Reconstructive Surgery, Sushrut Institute of Plastic Surgery, Lucknow, IND; 4 Department of Plastic Surgery and Reconstructive Surgery, King George's Medical University, Lucknow, IND; 5 Department of Plastic Surgery, King George's Medical University, Lucknow, IND; 6 Stem Cell Research Center, Department of Hematology, Sanjay Gandhi Postgraduate Institute of Medical Sciences (SGPGIMS), Lucknow, IND; 7 Department of Biotechnology, Swami Vivekanand Subharti University, Meerut, IND

**Keywords:** genetic polymorphisms, north indian population, non-syndromic patients, mthfr gene, cleft lip and palate

## Abstract

Introduction: Cleft lip and palate (CLP) is a common congenital anomaly characterized by incomplete fusion of the lip and/or palate during embryonic development. The etiology of CLP is multifactorial, involving genetics and different environmental factors. The methylenetetrahydrofolate reductase (*MTHFR*) gene has been proposed as a candidate gene associated with CLP due to its involvement in folate metabolism and DNA methylation processes. However, the association between *MTHFR* gene variants and CLP in non-syndromic patients in the North Indian population remains unclear.

Aim and objectives: This research aimed to see the association between *MTHFR* gene polymorphisms in non-syndromic patients with CLP in the North Indian population.

Materials and method: A case-control observational design comprised 50 CLP patients (cases) and 50 healthy individuals without CLP (controls). Blood samples were collected from patients visiting two hospitals. Genomic DNA was extracted from collected peripheral blood samples, and the genotyping of *MTHFR* gene polymorphisms (specifically, C677T) was performed using polymerase chain reaction-restriction fragment length polymorphism (PCR-RFLP) analysis. The allelic and genotypic frequencies of *MTHFR* gene variants were compared between cases and controls using appropriate statistical tests.

Result: This research revealed a significant association between* MTHFR* gene polymorphism and CLP in the North Indian population.

The odds for the genotypes reach statistical significance, suggesting that the *MTHFR* gene variant may play a major role in this population's susceptibility to non-syndromic CLP.

Conclusion: This study provides evidence for a linkage between the *MTHFR* gene C677T polymorphism and an increased risk of CLP in non-syndromic patients in the North Indian population. These findings do support the involvement of *MTHFR* gene variants in the etiology of CLP. In the future, more research is warranted to elucidate the underlying mechanisms linking *MTHFR* gene variants to CLP and to explore potential gene-environment interactions in this context.

## Introduction

Cleft lip and palate (CLP) is one of the most common congenital anomalies affecting the orofacial region [1]. It is characterized by the incomplete fusion of the lip and/or palate during embryonic development, leading to functional, aesthetic, and psychosocial challenges for affected individuals. CLP is a complex disorder that is influenced by a combination of different genetic and environmental factors [2]. Among the genetic factors, the methylenetetrahydrofolate reductase (*MTHFR*) gene has gained considerable attention due to its role in folate metabolism, DNA synthesis, and methylation processes [3], which involves homocysteine conversion to methionine.

The *MTHFR* gene is located on chromosome 1p36.3, i.e., at the short arm of chromosome 1 at 36 positions from centromere, and codes for an enzyme that plays a crucial role in the conversion of 5,10-methylenetetrahydrofolate to 5-methyltetrahydrofolate, which is necessary for the production of S-adenosylmethionine (SAMe), a major methyl donor in various biochemical reactions. Polymorphisms in the *MTHFR* gene, particularly the C677T (rs1801133) and A1298C (rs1801131) variants, have been extensively studied and have shown associations with a range of health conditions, including neural tube defects (NTDs), cardiovascular diseases, and certain types of cancer [4].

Several researchers have investigated the potential association between *MTHFR* gene polymorphisms and the risk of CLP, but the findings have been inconsistent, likely due to population-specific genetic variations [5-7]. The North Indian population, with its unique genetic background and distinct cultural and environmental factors, remains understudied in the context of *MTHFR* gene variants and their association with CLP.

Understanding the genetic factors involved in CLP is essential for several reasons. Firstly, it can contribute and add to our existing knowledge of the developmental processes and molecular mechanisms underlying CLP pathogenesis. Secondly, it may help in identifying individuals who are genetically susceptible to CLP, enabling targeted preventive measures and genetic counseling. Furthermore, such knowledge can have implications for personalized treatment approaches, including preoperative planning, postoperative care, and long-term management. The findings from this study may also have broader implications for genetic research on CLP and related craniofacial anomalies. They can contribute to the increasing body of evidence on the genetic basis of CLP and pave the way for future investigations into the interactions between *MTHFR* gene variants and other genetic or environmental factors that may modulate CLP risk.

A functional variation of the *MTHFR* gene exists because of the C677T mutation. As a result, the enzyme's activity is decreased after heating, and homozygotes who carry this heat-labile variation have elevated plasma homocysteine and are at a higher risk of developing NTDs [8]. There have been several existing case-control studies that have sought to link this polymorphism to the etiology of clefts, but the results have not proven promising. When larger populations are explored, associations have only been discovered in small studies that lack support [9-12]. Therefore, the present research aims to examine the association between *MTHFR* gene polymorphisms in non-syndromic patients with cleft lip and palate from the North Indian population. Cleft lip with or without cleft palate patients are of two types, non-syndromic and syndromic. Non-syndromic cleft patients have no associated syndromes of the body, but syndromic ones have many types of syndromes of the body with clefts, e.g., Apert syndrome and Pierre Robin syndrome. By focusing on this specific population, we can gain insights into the genetic underpinnings of CLP and potentially identify markers that may contribute to risk assessment, prevention, and treatment strategies.

Clinical significance

Studying genetics in cleft lip and palate patients helps to know genetic mutation and polymorphism. We can track inheritance and susceptibility in families of CLP, and this helps to plan for alternatives. Maternal diagnosis of CLP can reduce the anxiety of the family, and they can better be prepared. Prior diagnosis of a child can help better management of the child after birth. Genetic engineering has added a new dimension to the treatment of genetic diseases, and it is only possible because of correct diagnosis. Future stem cell therapy with tissue engineering can provide some solutions to patients with CLP.

## Materials and methods

A case-control observational study design examined the association of *MTHFR* gene polymorphisms in non-syndromic patients with cleft lip and palate (CLP) in the North Indian population. Permission to carry out the study was obtained from the Institutional Ethics Committee of King George's Medical University, Lucknow, Uttar Pradesh (UP) (approval number: 110 ECM II A/P10). Informed consent was sought from each participant or their legal guardian. Participant information confidentiality was maintained during the study.

The participants were grouped into cases and controls. Patients with non-syndromic CLP were included as cases, and their diagnoses were made after thorough clinical evaluations that included physical examinations and records reviewed by two plastic surgeons. Individuals with syndromic forms of CLP (e.g., eye, brain, limb anomalies, and cardiac defects) were excluded. People without a history of CLP or any other craniofacial deformities served as controls. Controls and cases were recruited from King George's Medical College and Sushrut Institute of Plastic Surgery, Lucknow, UP, where patients from Northern India visit for their treatment.

Clinical evaluation

Thorough clinical examinations were carried out to support the non-syndromic CLP diagnosis. This involved reviewing medical data and having patients undergo physical examinations by two plastic surgeons. Subjects were from nearby places of Lucknow, UP. The education status of most of the parents was below eighth standard, and most of them were economically poor. Marriages were non-consanguineous in most of the parents of CLP cases. The relevant information was that in most of the CLP cases, parents used Chula (smoke) as a cooking medium.

Sample collection

About 3 mL of peripheral venous blood from the median cubital vein was collected from each case and control in aseptic conditions in purple vacutainers, which contained ethylenediaminetetraacetic acid (EDTA) for the prevention of blood clotting. Collected blood samples were preserved at 4°C to avoid any hemolysis of RBCs till genomic DNA was extracted from each collected blood sample.

Genomic DNA extraction from blood

Using Qiagen kits, DNA was extracted from the acquired blood samples as per the manufacturer's guidelines. Polymerase chain reaction (PCR) and restriction fragment length polymorphism (RFLP) analysis were done to evaluate the association. Quality control measures were implemented to ensure accurate and reliable genotyping results. The ratio of sample absorbance at 260 nm and 280 nm was used to assess the purity of DNA (normal value: ≈1.8). A lower 260:280 ratio may indicate the presence of protein, phenol, and other contaminants (absorbance at 280 nm). To check if nucleic acid was free from salts, we used a ratio of sample absorbance at 260 nm and 230 nm (normal range: 1.8-2.2). A lower 260:230 ratio may indicate the presence of salts (absorbance at 230 nm).

PCR procedural steps

Primer Design

Specific primers are designed to target the regions of interest within the *MTHFR* gene that contain the desired single-nucleotide polymorphisms (SNPs). The primers are designed to specifically bind to the complementary sequences flanking the SNP sites. Hinf1 was the restriction enzyme used for digestion at the SNP site. The forward primer sequence is MTHFR HinF F- CTGGATGGGAAAGATCCCG, with a length of 19 base pairs, molecular weight (MW) of 5877.85, and guanine-cytosine percentage (GC%) of 57.89. The reverse primer sequence is MTHFR HinF R- CAGGTTACCCCAAAGGCCAC, with a length of 20 base pairs, MW of 6055.96, and GC% of 60.

PCR Steps and Conditions

Initial denaturation was done at 94°C for three minutes and then denaturation at 94°C for 40 seconds. The annealing step was carried out at 65°C for 30 seconds (35 cycles). Extension of DNA at 72°C was performed for one minute. The final extension step at 72°C was carried out for five minutes. The composition of the PCR master mix is 0.05 U/µL Taq DNA polymerase, reaction buffer, 4 mM Mgcl2, and 0.4 mM of each deoxynucleotide triphosphate (dNTP) (deoxyadenosine triphosphate (dATP), deoxycytidine triphosphate (dCTP), deoxyguanosine triphosphate (dGTP), and deoxythymidine triphosphate (dTTP)).

Protocol

The PCR master mix was gently vortexed and then briefly centrifuged after thawing. Then, a thin-walled PCR tube was placed on ice, and the following components for each 50 µL reaction were added: 25 µL PCR master mix, 0.5 µM forward primer, 0.5 µM reverse primer, 100 µg template DNA, and 50 µL nuclease-free water. Then, the samples were gently vortexed and spun. A DNA thermal cycler (SureCycler 8800, Agilent, Santa Clara, CA) using primer sequences was used to amplify the genomic DNA. The PCR amplified product of 5 µL was examined by electrophoresis on 2% agarose gel stained with ethidium bromide.

Restriction Digestion

After PCR, the following reaction mixture was prepared for RFLP analysis: 10 µL amplified PCR product, 1 µL restriction enzymes, 2 µL restriction enzyme buffer, and 7 µL double distilled water (total reaction volume: 20 µL). The reaction volume was incubated at a particular temperature depending upon the restriction enzyme, in case of fast digestion at 37°C for 15 minutes. The digested products were run on 3% agarose gel, and the size of the fragments was determined with the help of a ladder loaded on the gel.

Gel Electrophoresis

**Figure 1:**
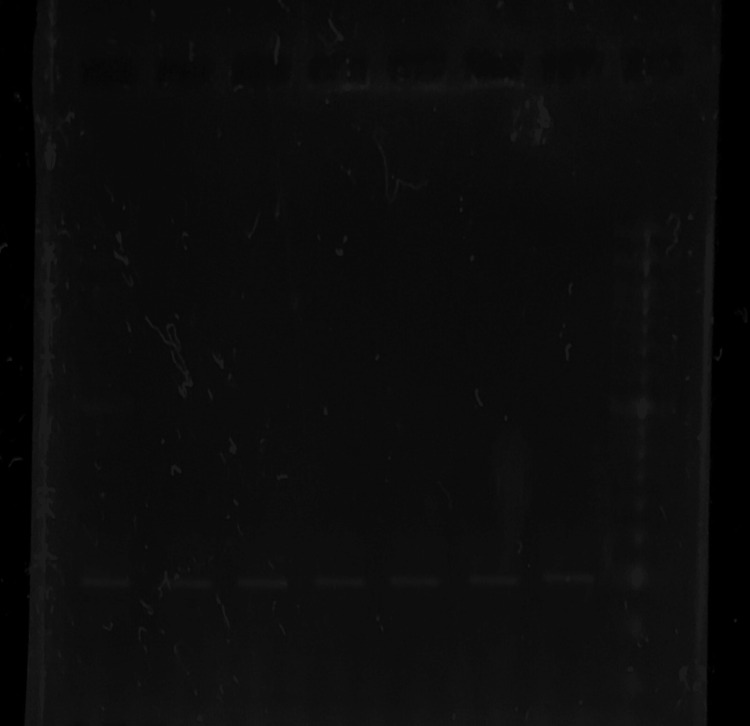
PCR product of 198 base pairs in the control group on gel PCR: polymerase chain reaction

**Figure 2:**
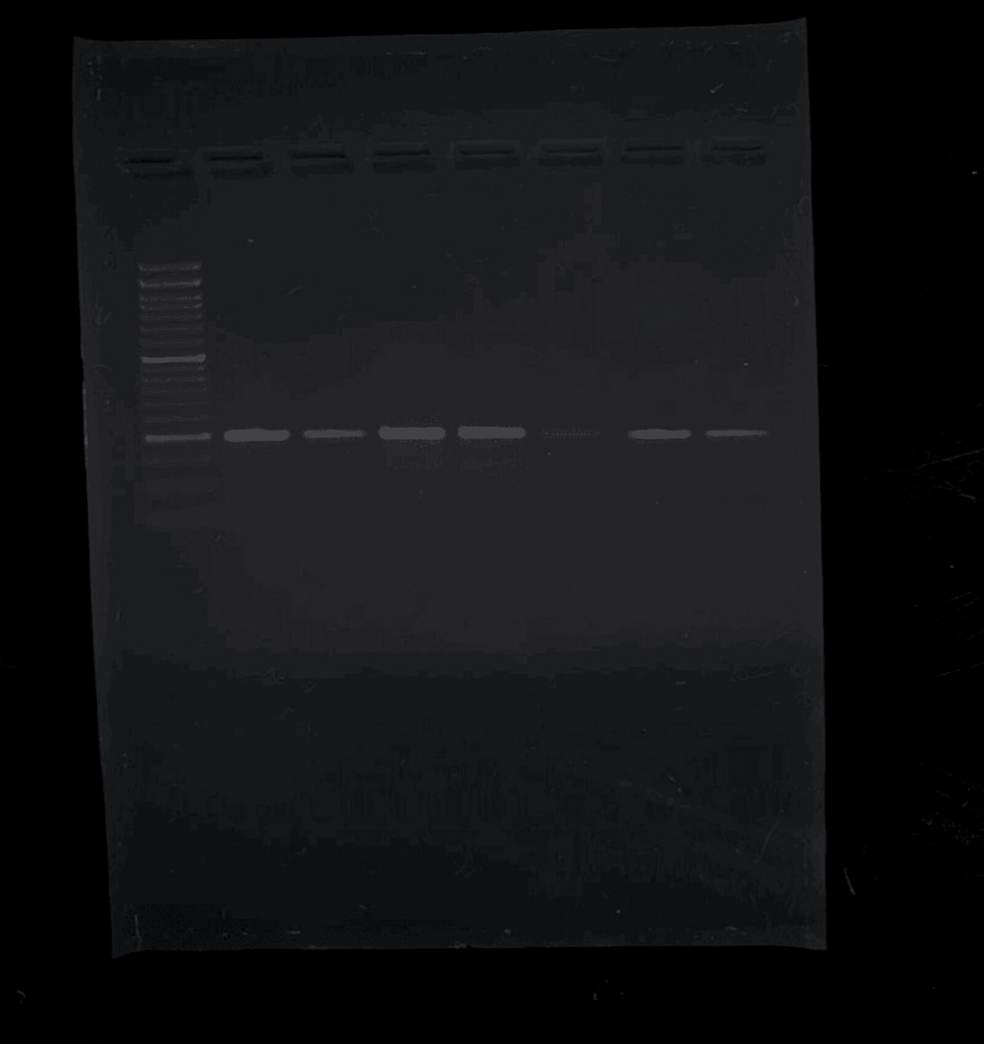
PCR product of 198 base pairs in the case group on gel PCR: polymerase chain reaction

Following PCR amplification, the resulting DNA fragments are visualized and analyzed using gel electrophoresis. The PCR products are separated based on size by running them through an agarose gel using an electric current. The gel is stained with a DNA-specific dye, and the fragments of DNA are visualized under ultraviolet light in a gel imager as shown in Figure 1 and Figure 2. The PCR product obtained was 198 bp for the control and case group and is depicted in Table 1 and Table 2.

**Table 1:** PCR product size of 198 bp A PCR product size of 198 bp is obtained in the control group of samples from 25 to 31. A 50 bp ladder is used in lane 8 to evaluate band size. PCR: polymerase chain reaction, bp: base pair

Control code	Lane	Band sizes
25	1	198 bp
26	2	198 bp
27	3	198 bp
28	4	198 bp
29	5	198 bp
30	6	198 bp
31	7	198 bp
Ladder	8	50 bp

**Table 2:** PCR product size of 198 bp A PCR product size of 198 bp is obtained in the case group of samples from 11 to 17. A 50 bp ladder is used in lane 1 to evaluate band size. PCR: polymerase chain reaction, bp: base pair

Case code	Lane	Band size
Ladder	1	50 bp
P11	2	198 bp
P12	3	198 bp
P13	4	198 bp
P14	5	198 bp
P15	6	198 bp
P16	7	198 bp
P17	8	198 bp

**Figure 3:**
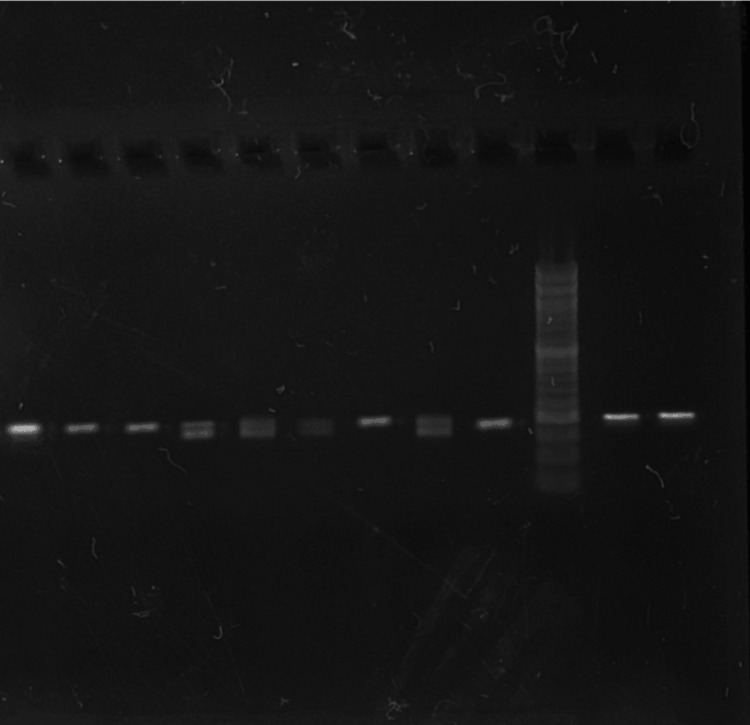
Restriction digestion with HinF1 enzyme in the control group on gel

**Figure 4:**
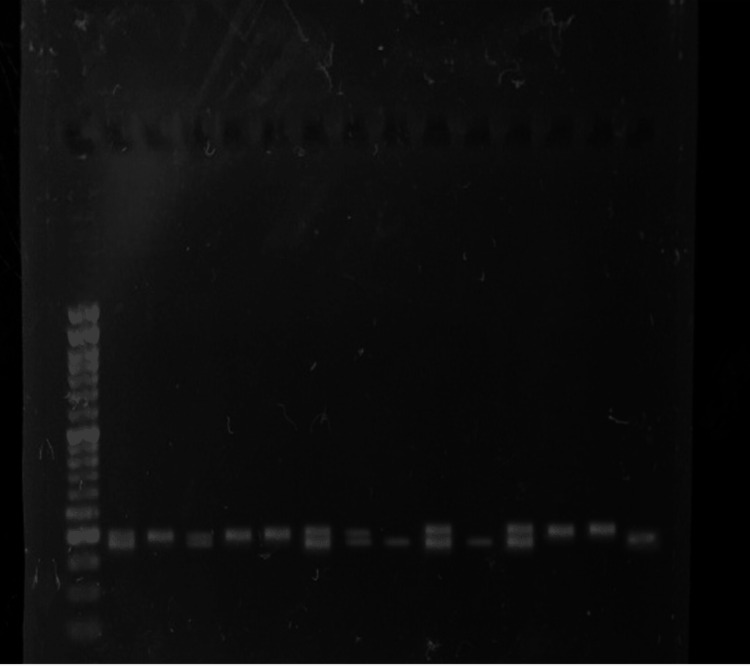
Restriction digestion with HinF1 enzyme in the case group on gel

After this, the PCR product was digested with the help of the restriction digestion enzyme HinF1. By comparing the size of the bands on agarose gel with a known loaded ladder, the presence of band size was calculated as shown in Figure 3 and Figure 4. If there was digestion, then there was a mutation, and if no digestion happened, then there was no mutation, and it was termed as a wild type both in the control and case groups as shown in Table 3 and Table 4. The presence or absence of specific genetic variations, such as the *MTHFR* gene polymorphisms, was determined: wild, band size of 198 bp on the gel; heterozygous mutant, band sizes of 198 bp, 175 bp, and 23 bp on the gel; and homozygous mutant, band sizes of 175 bp and 23 bp on the gel.

**Table 3:** Band sizes after restriction digestion Restriction digestion of PCR product of the control group of samples 23-33 and bands of 198, 175, and 23 bp are obtained. A comparison ladder of 50 bp is used in lane 10 to evaluate band sizes. PCR: polymerase chain reaction, bp: base pair, H: heterozygous, M: homozygous mutant, W: wild type

Control code	Lane	Result	Number of bands	Band sizes
23	1	W	1	198 bp
24	2	W	1	198 bp
25	3	W	1	198 bp
26	4	H	3	198, 175, and 23 bp
27	5	H	3	198, 175, and 23 bp
28	6	H	3	198, 175, and 23 bp
29	7	W	1	198 bp
30	8	H	3	198, 175, and 23 bp
31	9	M	2	175 and 23 bp
Ladder	10	-	-	50 bp
32	11	W	1	198 bp
33	12	W	1	198 bp

**Table 4:** Band sizes after restriction digestion Restriction digestion of PCR product of the case group of samples 11-24 and bands of 198, 175, and 23 bp are obtained. A comparison ladder of 50 bp is used in lane 1 to evaluate band sizes. PCR: polymerase chain reaction, bp: base pair, H: heterozygous, M: homozygous mutant, W: wild type

Case code	Lane	Result	Number of bands	Band sizes
Ladder	1	-	-	50 bp
11	2	H	3	198, 175, and 23 bp
12	3	W	1	198 bp
13	4	H	3	198, 175, and 23 bp
14	5	W	1	198 bp
15	6	W	1	198 bp
16	7	H	3	198, 175, and 23 bp
17	8	H	3	198, 175, and 23 bp
18	9	M	2	175 and 23 bp
19	10	H	3	198, 175, and 23 bp
20	11	M	2	175 and 23 bp
21	12	H	3	198, 175, and 23 bp
22	13	W	1	198 bp
23	14	W	1	198 bp
24	15	M	2	175 and 23 bp

Outcomes evaluated

In the context of this study, "heterozygous mutant," "wild-type," and "homozygous mutant" are used to describe different genetic variants or genotypes. For the present study, only the C677T variant was considered. The C677T variant refers to a specific SNP in the *MTHFR* gene. This variant occurs at position 677 of the gene's coding sequence. The *MTHFR* gene is located on chromosome 1p36.3 and consists of 11 exons. The C677T variant specifically occurs in exon 4 of the *MTHFR* gene.

Heterozygous Mutant

Heterozygous refers to a genetic condition where an individual carries two different alleles (variants) of a particular gene at a specific locus. A heterozygous genotype for the C677C would indicate that an individual has inherited one copy of the "C" allele and one copy of the "T" allele at that specific position on the gene.

Wild Type

The term "wild-type" refers to the most common or prevalent form of a gene or allele in a population. In the context of the *MTHFR* gene, the wild-type genotype for the C677T is CC, indicating that both copies of the gene carry the "C" allele, i.e., C677C.

Homozygous Mutant

The term "mutant" refers to a genetic variant that deviates from the wild-type allele. In the case of the *MTHFR* gene, the mutant genotypes for the C677T include TT, indicating the presence of two copies of the "T" allele.

The genotyping analysis of the *MTHFR* gene in the study of CLP in our study involved determining the presence or absence of specific alleles or genotypes, including heterozygous, wild-type, and mutant genotypes. By comparing the frequencies of these genotypes between the cases with CLP and control individuals, we tried to evaluate the potential association between specific *MTHFR* genotypes and the risk of CLP in the studied population.

## Results

The current study investigated the association between the *MTHFR* gene polymorphism and the occurrence of cleft lip and palate (CLP) in non-syndromic patients in the North Indian population. Genotyping analyses were performed to see the distribution of *MTHFR* gene polymorphisms in both CLP cases and control individuals. *MTHFR* genotypes (H, M, and W) were summarized in number (n) and percentage (%) and compared using the chi-square (χ2) test. The genotypes were in Hardy-Weinberg equilibrium (HWE). A two-tailed (α=2) P<0.05 was considered statistically significant. The analysis was performed on SPSS software version 21 (IBM SPSS Statistics, Armonk, NY).

**Figure 5:**
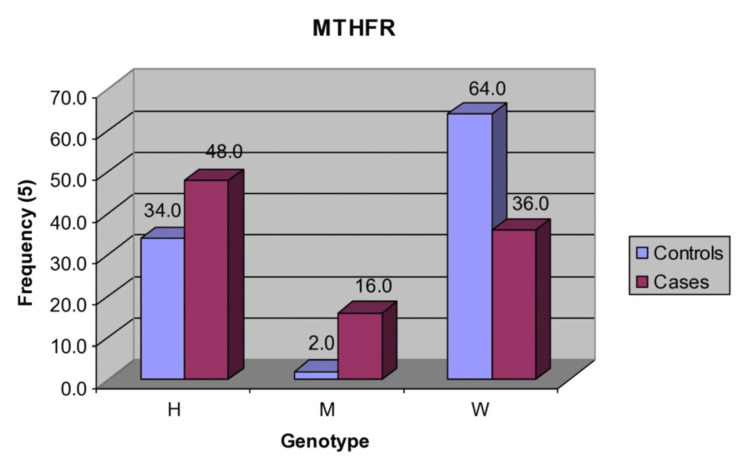
Frequency distribution of MTHFR genotypes in cases and controls *MTHFR*: methylenetetrahydrofolate reductase, H: heterozygous, M: homozygous mutant, W: wild type

A total of 100 subjects, 50 healthy controls free from any defect and 50 cases with non-syndromic cleft lip and palate from the north Indian population, were randomly selected from the same hospitals. The *MTHFR* genotypes (H, M, and W) of the two groups are summarized in Table 5 and also depicted in Figure 5. In controls, there were 17 (34%) heterozygous, one (2%) homozygous mutant, and 32 (64%) wild type. In contrast, in cases, there were 24 (48.05%), eight (16%), and 18 (36%), respectively. The frequency (%) of heterozygous was 14% (or 1.4-fold) more in cases as compared to controls. Furthermore, the distribution of homozygous mutants was also found to be 14% (or 8-fold) more in cases than controls. However, the frequency of wild type was 28% (or 1.8-fold) more in controls as compared to cases.

**Table 5:** Frequency distribution of MTHFR genotypes in cases and controls Genotypes of two were summarized in number (n) and percentage (%) and compared using the χ2 test. *P<0.01 = significant, P>0.01 = non-significant *MTHFR*: methylenetetrahydrofolate reductase, H: heterozygous, M: homozygous mutant, W: wild type

Genotype	Controls (n=50) (%)	Cases (n=50) (%)	χ^2^ value	P value
H (allele CT)	17 (34)	24 (48)	10.56	0.005^*^
M (allele TT)	1 (2)	8 (16)		
W (allele CC)	32 (64)	18 (36)		

Comparing the frequency of *MTHFR* genotypes (H, M, and W) of the two groups, the χ2 test showed significantly (P<0.01) different genotypes between the two groups (χ2=10.56, P=0.005). In conclusion, heterozygous and homozygous mutants both accounted for significant and 28% (or 1.8-fold) higher frequencies in cases as compared to controls.

The higher frequencies/prevalence of both heterozygous and homozygous mutants of the *MTHFR* gene in cases is responsible for CLP occurrence; thus, the *MTHFR* gene may be a genetic marker for CLP in the North Indian population.

## Discussion

The present research aimed to investigate the association between the *MTHFR* gene and the occurrence of cleft lip and palate in non-syndromic patients within the North Indian population. The findings provide valuable insights into the genetic factors contributing to this common craniofacial anomaly and have important implications for understanding its etiology and potential preventive strategies.

The findings of our study revealed a significant association between the *MTHFR* gene variant C677T and cleft lip and palate in the North Indian population. Specifically, we observed different distributions of the C677T variant in the case group and the control group. This suggests that individuals carrying the C677T variant may have an increased susceptibility to developing cleft lip and palate. However, certain studies' findings are not consistent with our study findings, which were conducted in different populations, highlighting the insignificant role of the *MTHFR* gene in the etiology of this condition. Zhao et al. [13] observed no association between *MTHFR* polymorphism and non-syndromic cleft lip with or without palate (NSCL/P) risk in their meta-analysis that included nine case-control studies evaluating the Asian population. Similar observations were noted in the study of Verkleij-Hagoort et al. [14], and Johnson and Little [15] reported no associations between *MTHFR* C677T and A1298C polymorphisms and NSCL/P.

Some observations were also made in this context that support our study findings. Luo et al. [16] indicated that the maternal *MTHFR* 677TT genotype was inclined with a greater chance of having an NSCL/P offspring. However, another quantitative synthesized analysis conducted by Pan et al. [17] showed that *MTHFR* C677T polymorphism attributed to an elevated risk of NSCL/P among Asians.

The *MTHFR* gene encodes an enzyme involved in folate metabolism, which is critical for normal embryonic development, including proper closure of the lip and palate. The C677T variant has been shown to affect *MTHFR* enzyme activity, leading to alterations in folate metabolism and potentially compromising embryonic development [18]. Thus, the hypothesis is that genetic variations in the *MTHFR* gene contribute to the development of cleft lip and palate.

The C677T variant refers to a SNP in the *MTHFR* gene. This variant occurs at position 677 of the gene's coding sequence. The *MTHFR* gene is located on chromosome 1p36.3 and consists of 11 exons. The C677T variant specifically occurs in exon 4 of the *MTHFR* gene. It involves a substitution of the cytosine (C) nucleotide at position 677 with a thymine (T) nucleotide in the gene's DNA sequence. Exons are the regions of a gene that contain the instructions for protein synthesis [19,20].

The C677T variant can affect the activity of the methylenetetrahydrofolate reductase enzyme, which is encoded by the *MTHFR* gene. Our study results are not in correlation to other Indian studies by Abdulla et al. [21] and Desai et al. [22], which were mainly done for the South Indian population. Individuals with the C677T genotype may have decreased enzyme activity, potentially leading to changes in folate metabolism and associated health outcomes. The C677T variant has been studied extensively, and it has been associated with various health conditions. For example, individuals with the C677T homozygous genotype (TT) may have 70% reduced enzyme activity compared to those with the wild-type genotype (CC) and a 35% decrease for the heterozygous genotype. This reduced enzyme activity can affect folate metabolism and is linked with an increased risk of certain health conditions, including neural tube defects, cardiovascular disease, and psychiatric disorders.

However, it is important to acknowledge certain limitations of our study. The sample size was relatively small, which may limit the generalizability of our findings to the entire North Indian population. Further studies with larger sample sizes are needed to confirm our results and provide more robust evidence. We studied the North Indian population in our study and enquired about each case and control for their past three generations only. However, there is a likely chance that earlier generations may have migrated from other regions and settled here. To overcome this, we need to enquire about the history of many generations of each mother and father of all the cases and controls and extract the pure North Indian population. Therefore, future research with larger sample sizes and multi-ethnic populations is warranted to validate these results and explore potential gene-environment interactions that may influence CLP risk.

## Conclusions

In conclusion, we can say that our research provides evidence of an association between the *MTHFR* gene and the occurrence of cleft lip and palate in non-syndromic patients within the North Indian population. These observations contribute to our understanding of the genetic basis of this craniofacial anomaly and have potential implications for risk assessment, genetic counseling, and preventive strategies. Further research is warranted to validate our results, investigate gene-environment interactions, and explore the underlying biomolecular mechanisms involved in the development of cleft lip and palate. Ultimately, a better understanding of the genetic factors contributing to this condition can aid in early detection, intervention, and improved outcomes for affected individuals and their families.
